# Assessment by Women on Selected Aspects of Quality of Life and on Disease Acceptance after Undergoing Urogynecological Procedures for Urinary Incontinence

**DOI:** 10.3390/jcm12154894

**Published:** 2023-07-26

**Authors:** Bożena Baczewska, Katarzyna Wiśniewska, Bożena Muraczyńska, Małgorzata Pasek, Jolanta Słuszniak, Katarzyna Gębicka, Beata Guzak

**Affiliations:** 1 Department of Internal Medicine and Internal Medicine in Nursing, Faculty of Health Sciences, Medical University of Lublin, Chodźki 7, 20-093 Lublin, Poland; 2 Faculty of Health Sciences, Radom College, 1905 Roku 26/28, 26-600 Radom, Poland; 3 Faculty of Medical Sciences and Health Sciences, University of Natural Sciences and Humanities, Konarskiego 2, 08-110 Siedlce, Poland; 4 Department of Nursing, Faculty of Health, University of Applied Sciences in Tarnów, 33-100 Tarnów, Poland; 5 The J. Dietl Specialist Hospital, Skarbowa 4, 31-121 Kraków, Poland; 6 Department of Pharmaceutical Microbiology, Medical University of Lublin, Chodźki 1, 20-093 Lublin, Poland; 7 Center of Postgraduate Education for Nurses and Midwives, Pawińskiego 5A, 02-106 Warsaw, Poland

**Keywords:** urinary incontinence, urologic surgical procedures, quality of life

## Abstract

Urinary incontinence is a common social and health problem that affects both men and women. Women, however, are twice as likely as men to experience unintentional and involuntary bladder emptying due to their anatomical structure and biological functions. Urinary incontinence is associated with great discomfort, a sense of shame, and a significant reduction in self-esteem, often resulting in the limiting of, or withdrawing from, professional, social, and community life. The aim of this study was to evaluate selected aspects of the quality of life and disease acceptance by women who had undergone urogynecological procedures for urinary incontinence. The study encompassed 77 women. The diagnostic tools used in the study were the Polish versions of the King’s Health Questionnaire (KHQ), the Acceptance of Illness Scale (AIS), and the authors’ own survey questionnaire. From the undertaken research, we found that women with urinary incontinence who underwent urogynecological procedures rated their overall health well. What worsened the most regarding the quality of life of women post-procedure for urinary incontinence was the impact of bladder problems on their lives and the discomfort they felt due to bladder problems. The relationship between the time elapsed since the procedure and the quality of life of the respondents with regard to the emotions they experienced was also found to be significant. The longer the time since the procedure, the lower the intensity of negative emotions, and thus the higher the quality of life. Despite the varied opinions of the respondents about the impact of bladder dysfunction on various areas of their lives, acceptance of the disease, as measured by the AIS scale, appeared to be high.

## 1. Introduction

Urinary incontinence is considered one of the most important health problems of the 21st century and one of the most common chronic conditions in women. Both the prevalence and severity of incontinence problems increase with age [1]. The estimated number of people with the condition worldwide is about 423 million. In Poland, the problem affects more than 2–3 million people. It is believed that the percentage may be higher due to the intimate nature of the condition, which makes it difficult to collect reliable data associated with social stigma [2,3]. Many women with incontinence do not dare to admit the problem even to their gynecologist, or they only report it in the second or third stage of the disease, when the condition qualifies for drug treatment or surgical intervention [4,5,6,7].

Numerous studies [1,5,6,7,8,9,10,11] show that urinary incontinence significantly reduces the quality of life in the biological, psychological, and social spheres. Other studies, the literature of which was reviewed [12], and from which literature a total of 23 articles, with 24,983 respondents, mostly women, was examined, also show that incontinence is associated with poor quality of life. According to the World Health Organization and the International Continence Society (ICS), urinary incontinence is defined as the involuntary, uncontrolled leakage of urine from the bladder through the urethra [13]. This phenomenon affects both men and women regardless of age. However, women are affected twice as often as men due to biological functions, as well as the anatomical structure of the urethra [3]. The first peak in incidence is observed in menopausal women, while an upward trend is seen in women over the age of 65. Globally, an average of about 30–40% of all women struggle with urinary incontinence before menopause and up to 60% after menopause [14,15].

The problem of incontinence is not new, but despite this, there is still a perception in society that the disease is embarrassing. It is often the case that sufferers do not report the ailment to a doctor for many years or do not do so at all. Studies show that a culture of secrecy and deep shame constitutes a barrier to seeking help. Urinary incontinence is sometimes associated with great discomfort, embarrassment and a significant reduction in a woman’s self-esteem [5]. For this reason, there is often a reduction or abandonment of professional, social, and community life, and the consequent appearance of depressive symptoms [2,3]. The World Health Organization defines quality of life as an individual’s perception of his or her position in life within the cultural context and value system in which he or she lives and in relation to the tasks, expectations, and standards set by environmental conditions [16]. Based on research [10,17,18], it appears that urinary incontinence significantly reduces a woman’s quality of life.

Treatment methods for incontinence can be broadly divided into conservative, among which physiotherapy is recommended as the first line of therapy due to the low cost and low risks associated with it, pharmacological, and surgical. Frequently used conservative treatments include pelvic floor muscle training (especially important in women with stress urinary incontinence), electrostimulation, and behavioral therapy to change urination behavior. For example, research by Mikuš M. et al. [19] shows the effectiveness of the EMI (Extracorporeal Magnetic Innervation) method in comparison with Kegel exercises. Patients treated with EMI have fewer episodes of urinary incontinence and better quality of life. In turn, the study by Frutos-Reoyo E.J. et al. from 2023 [20] indicates the effectiveness of rehabilitation procedures such as kinesitherapy or electrical stimulation of the posterior tibial nerve. The correct choice of treatment depends largely on early diagnosis, the patient’s condition, and the type and degree of incontinence. Surgical treatment is introduced after conservative treatment options have been exhausted and is mainly used in cases with a significant degree of incontinence [21].

The main objective of the study was to study the evaluation by women who had undergone urogynecological procedures for urinary incontinence of selected aspects of the quality of life, as well as disease acceptance.

## 2. Materials and Methods

The Polish version of the King’s Health Questionnaire (KHQ) [22], the Acceptance of Illness Scale (AIS) [23], and the authors’ own survey questionnaire were used for the study. The first part of this questionnaire contained questions on sociodemographic variables, while the second part of the questions concerned clinical variables. In the part of the questionnaire concerning clinical variables, the respondents were asked about the duration of the illness, the time since the urogynecological procedure, the type of urinary incontinence, the type of urogynecological procedure performed, etc.

The King’s Health Questionnaire (KHQ) is used to measure the level of quality of life for those with incontinence-related conditions. The questionnaire contained 32 closed-ended questions that assessed the quality of life in nine domains, such as general health assessment, the impact of incontinence on daily life, ADL (Activities of Daily Living) limitations, physical and social limitations, limitations on personal life, impact on perceived emotions, impact on sleep and vitality, and measures of incontinence severity. The higher the final score obtained, the lower the subjects’ quality of life and the more severe the problems resulting from incontinence. The second tool used in the study was the Acceptance of Illness Scale (AIS) questionnaire. The scale contained eight statements to which the respondents answered on a five-point Likert scale, where 1 meant poor adaptation to the disease, while 5 meant full acceptance of the disease. Total scores ranged from 8 to 40, and the higher the total score, the greater the acceptance of the disease.

**Figure 1:**
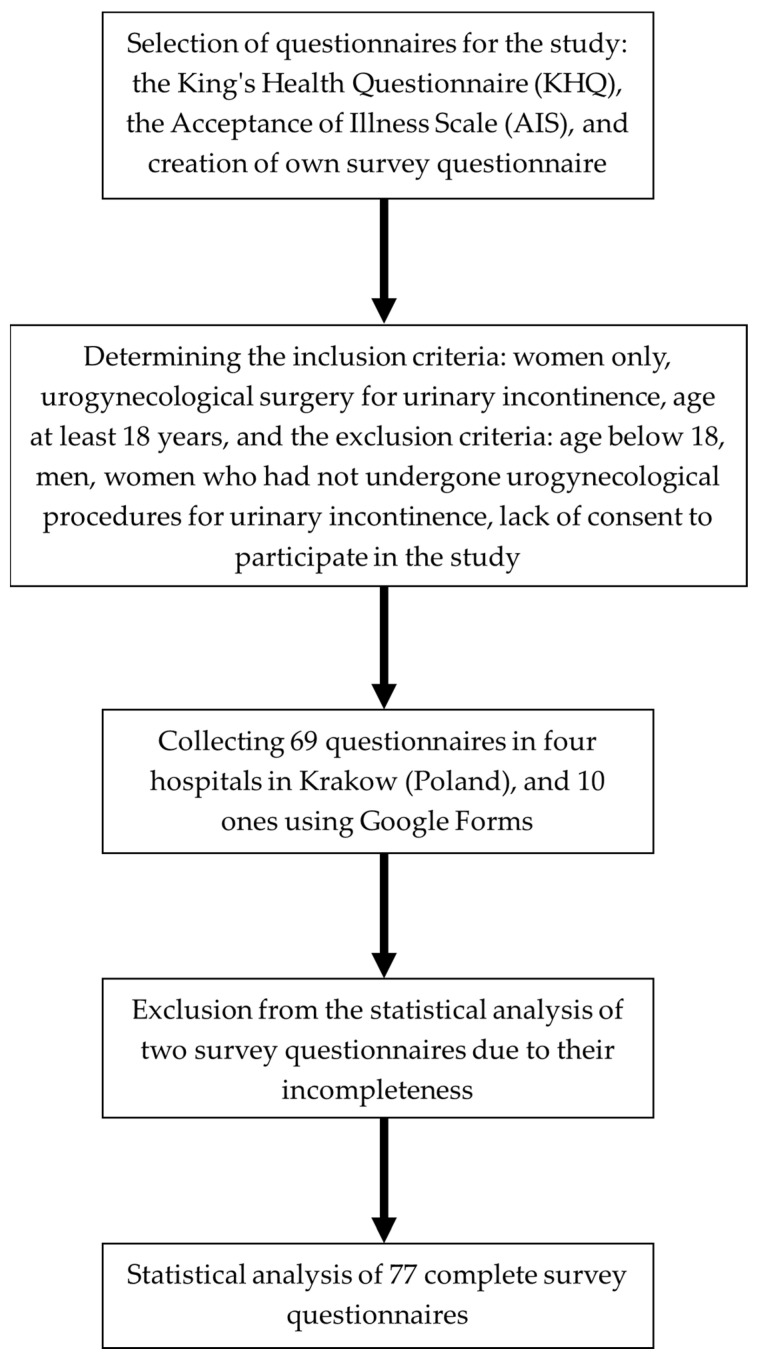
Flowchart of conducting the study.

The inclusion criteria for the study were women who had undergone surgery for urinary incontinence and were at least 18 years old. The exclusion criteria included age below 18, men, women who had not undergone urogynecological procedures for urinary incontinence, and lack of consent to participate in the study. All respondents were informed of the confidentiality of their personal information and gave informed consent to participate in the study. The study was conducted in the first quarter of 2021 in Poland, in four hospitals in Krakow (69 surveys), and some of the survey questionnaires were distributed in an electronic version via the Google Forms platform (10 surveys). In the process of qualifying the questionnaires for statistical analysis, two were rejected in their entirety due to incompleteness. The course of the study is presented in Figure 1.

The study encompassed 77 women that underwent urogynecological procedures for urinary incontinence, ranging in age from 24 to 82 years. The mean age was 54.46 ± 12.20 years. The vast majority of the respondents were married (n = 59; 76.62%). The mean time since surgery was 4.35 ± 4.82 years, while the mean duration of illness was 5.04 ± 3.32 years. All study participants had given birth, most of them by natural force (n = 62; 80.52%). More than half of the respondents experienced stress urinary incontinence (n = 43; 55.84%), 20 (25.97%) of the women had mixed incontinence, while 4 (5.19%) of the women had symptoms of overactive bladder (urge incontinence). Women most often underwent TVT (tension free vaginal tape) sling, 32.47%, and TOT (transobturator tape) sling, 29.87%.The characteristics of the respondents are presented in Table 1.

### Statistical Analysis

Descriptive statistics were used to describe the collected data, and counts and percentages were calculated. The Kruskal–Wallis test, the Mann–Whitney U test, and Spearman’s rank correlation coefficient were used in the statistical analysis. The Kruskal–Wallis test was used when comparing more than two independent samples, the Mann–Whitney U test was applied when comparing two independent samples, and the Spearman rank correlation coefficient was employed when examining the relationship between quantitative data. Non-parametric statistics were used due to the fact that the distribution of variables differed significantly from normal, which was confirmed by the Shapiro–Wilk W test. A significance of differences and correlations was found at *p* < 0.05.

## 3. Results

### 3.1. The KHQ Results

The assessment of nearly half of the respondents about their overall health after their urogynecological procedures for urinary incontinence was good (n = 36; 46.75%). More than a quarter of the women rated their health as satisfactory (n = 22; 28.57%), and the remaining women rated their health as very good (n = 16; 20.78%), while three women (3.90%), rated their overall health as poor. None of the women rated their health as very bad. The women’s assessment of the impact of bladder dysfunction on their daily lives varied. The majority of women said that continued incontinence problems, despite urogynecological surgery, affect their daily lives to a degree described as very, somewhat, and moderate, respectively: (n = 26; 33.77%), (n = 22; 28.57%), (n = 19; 24.68%). In contrast, 10 (12.99%) of the women said that their incontinence problem does not impact their daily life. The results of the study are shown in Table 2.

The enrolled also assessed the severity of their symptoms and the impact of the symptoms on their lives. For 8 (10.39%) women, frequent going to the toilet was felt quite strongly, while 38 (49.35%) women described the burdensomeness of such toilet visits as moderate. Nocturia was felt quite strongly by 7 (9.09%) women and as moderate, or average, by 28 (36.36%) respondents. Urge incontinence, and urine release associated with a strong need to urinate, was felt quite strongly by 13 (16.88%) women and as moderate by 23 (29.87%) of the respondents. Stress incontinence during physical activity, including coughing and sneezing, was experienced rather severely by 21 (27.27%) women and to a moderate degree by 12 (15.58%) of the respondents. However, it should be noted that a large percentage of women did not experience symptoms related to bladder disorders or experienced them at a ‘somewhat’ level. Detailed results in this regard are shown in Table 3.

Table 4 shows the impact of bladder dysfunction on daily activities, physical and social limitations, personal life, emotions, rest and well-being, and the occurrence of incontinence discomfort. Most often, the rating was somewhat, sometimes, or not at all. However, a quarter of women experienced a moderate impact of bladder dysfunction on traveling (24.68%), and a fifth on performing professional duties (work) and other activities outside the home (20.78%).

Perceptions of discomfort associated with incontinence, i.e., the need to wear sanitary pads, paying attention to the amount of fluids drunk, the need to change underwear when wet, unpleasant odor, and feelings of shame and embarrassment about bladder dysfunction, were rated differently by the respondents. Almost a third of the women (n = 23; 29.87%) constantly wore sanitary pads to keep their underwear dry, while 17 (22.08%) women did so frequently. The need to change underwear constantly when it becomes wet was reported by 23 (29.87%) female respondents and 20 (25.97%) of them changed their underwear frequently. Nearly a third of the respondents often paid attention to how much fluid they drank (n = 29; 37.66%), and 10 (12.99%) women constantly monitored their fluid intake. Nearly a quarter of the women were constantly concerned that they were emitting an unpleasant odor (n = 17; 22.08%), and nearly a third were frequently concerned about that (n = 24; 31.17%). Most of the women never felt embarrassed about their bladder problems or felt so only sometimes, respectively: (n = 21; 27.27%) and (n = 30; 38.96%).

The quality of life of the respondents was examined in 10 domains: self-assessment of general health, the impact of bladder problems on life, limitations in performing daily activities, physical limitations, social limitations, personal life, emotions, sleep/effort, measures of discomfort severity, and a symptom severity scale. The quality of life was most impaired by the impact of bladder problems on life (Me = 66.67) and the perceived discomfort from bladder problems (Me = 58.33). The results of the subjective assessment of the quality of life are shown in Table 5.

Statistical analysis showed a weak, negative correlation in the emotional domain between time since surgery and the quality of life of the women after urogynecological procedures. Herein, the longer the time since the procedure, the lower the intensity of negative emotions and the higher the quality of life of the respondents. A weak negative correlation between age and quality of life for women after urogynecological procedures was found in the case of self-assessment of general health and the domain pertaining to limitations in performing daily activities. Accordingly, the higher the age, the greater the severity of bladder problems and the lower the quality of life of the women. In the other categories, there was no significant relationship between time since surgery and age and the quality of life of the women after urogynecological procedures. The results of the statistical analysis are shown in Table 6.

### 3.2. The AIS Scale Results

**Figure 2:**
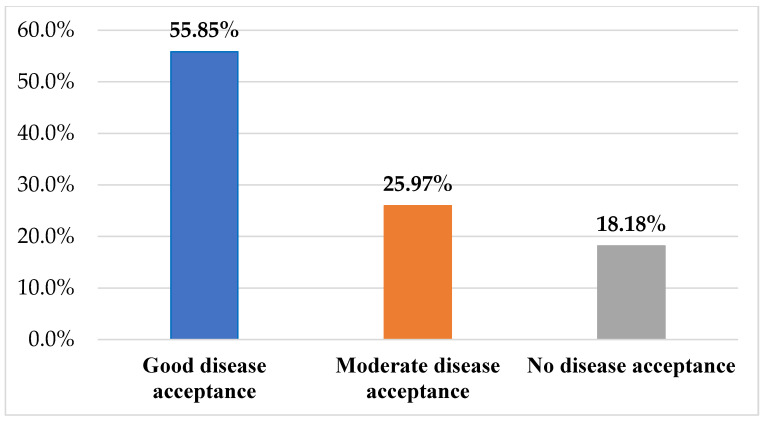
Levels of acceptance of the disease as measured by the AIS scale.

The women after urogynecological surgery also assessed the acceptance of their disease. The majority of surveyed women accepted their condition related to the disease at a good level (n = 43; 55.85%), while 20 women (25.97%) accepted it at an average level, and the remaining women did not accept their condition (n = 14; 18.18%). The results in this regard are illustrated in Figure 2.

The average level of acceptance of the disease was 29.06 ± 10.23 points, with half of the respondents having at least 32 points. In general, the degree of acceptance of the disease among the studied women who underwent urogynecological procedures for urinary incontinence, measured by the AIS scale, was found to be high. The results in this regard are shown in Table 7.

### 3.3. Correlation between the AIS Scale and the KHQ

Statistical analysis showed a significant relationship between acceptance of the disease as measured by the AIS scale and the quality of life of the women after urogynecological procedures in the domains studied. The higher the acceptance of the disease, the lower the severity of bladder problems and the higher the quality of life of the respondents. The relationship shown was quite weak in the domain of the self-assessment of the general health of the respondents, while it was moderate in the domains of limitations in performing daily activities, physical limitations, social limitations, sleep/effort, measures of discomfort severity, and the symptom severity scale, and strong for the domain involving emotions. A significant relationship was not shown only in the category involving the impact of bladder problems on life. Detailed results are presented below in Table 8.

## 4. Discussion

Choosing the right tool to assess the quality of life of women with urinary incontinence was a difficult task because researchers employ different instruments with different approaches [24,25,26,27,28,29]. In this study, the Polish version of the King’s Health Questionnaire (KHQ) was used. In the study by Kieres P. et al. [26], this enabled the researchers to obtain good psychometric values and, according to the authors of the study, is a useful diagnostic tool in the population of women with urinary incontinence. Our research has shown that women with urinary incontinence who underwent urogynecological procedures rate their QoL at different levels, considering different domains of life. Significant correlations were obtained in the domain of the impact of bladder problems on life (Me = 66.67) and in the domain of feelings of discomfort (Me = 58.33). Significant correlations were obtained between the age and quality of life of women who underwent urogynecological procedures in the case of the self-assessment of general health and the domain of limitations in performing daily activities. Herein, the higher the age, the greater the severity of bladder problems and the lower the quality of life of the women. Our research showed that more than half of the women did not have lower self-esteem (n = 47; 61.04%), but manifested both anxiety and depression at the ‘somewhat’ level, n = 37; 48.05% and n = 36; 46.75%, respectively.

Women after urogynecological procedures noted the need to constantly and frequently wear sanitary pads and to pay attention to the amount of fluids they drank, n = 40; 52.0% and n = 39; 51.0%, respectively. Statistical analysis showed a significant relationship, in the domain of emotions, between the time elapsed since urogynecological surgery and women’s quality of life. This is manifested as the longer the time since urogynecological surgery for urinary incontinence, the lower the intensity of negative emotions in women, and thus the higher quality of life.

Studies by other researchers [4,8,12,18,30] highlight the impact of bladder dysfunction on sexual life, resulting in the abandonment of sexual activity. These studies also show that people with incontinence often limit going out of the house and give up work and social contacts. Our research partially confirmed such a relationship, though the majority of women after urogynecological procedures for urinary incontinence had no problems with social gatherings or social contacts, respectively: n = 48; 62.34% and n = 45; 58.4%. Studies from earlier years [11], which included articles published between 2005 and 2010 and conducted among women with urinary incontinence, show that for many women, urinary incontinence is distressing and has a negative impact on health-related quality of life (HRQoL). Similar results were obtained by the authors of papers from recent years [5,6,7,12]. The authors of these studies emphasize that in assessing the quality of life of women with incontinence, different research tools should be used to obtain information about the diverse reactions to the condition by women. Our study shows that women who had undergone urogynecological procedures for urinary incontinence rated the overall state of their health as good (n = 36; 46.75%), satisfactory (n = 22; 28.57%), and very good (n = 16; 20.78%), although 3 (3.90%) women rated their health as poor.

In their research studies, Burzynski et al. [31] emphasize that sexuality plays a significant role at different stages of a woman’s life. Nevertheless, at each of these stages, sexuality is supposed to bring pleasure, gratification, fulfillment, and satisfaction. Loss of urine during sexual intercourse to a large extent contributes to a decrease in the frequency of intercourse, or even the abandonment of intercourse altogether. The most common factors limiting sexual activity in women with incontinence, the authors of this study write, are reduced libido, fatigue, lack of desire for sex, and lack of body acceptance. Faced with the mere awareness of losing urine very often leads to the abandonment of sexual activity. These studies also show that the most common ways women cope with losing urine during intercourse include urinating before intercourse, having intercourse only in safe places, limiting physical activity during intercourse, and reducing the frequency and duration of intercourse. Another study [32] of sexually active women with incontinence shows a similar result. These studies found that half of the women with incontinence who were surveyed felt that their sex life was more or less unsuccessful due to incontinence or urgency and that they feared urine leakage during intercourse, with nearly two-thirds of the subjects fearing an unpleasant odor and feeling uncomfortable and unattractive. In the same study, one-third of the respondents reported urine leakage during sexual activity. The dissatisfaction of women with their sex lives was strongly correlated with unsatisfactory mental health, incomplete orgasm, and the fear of urine leakage during intercourse. Insufficient vaginal lubrication, unsatisfactory mental health, and poor health were significantly correlated with decreased sexual desire.

Our research showed that more than half of the women who took part in our study, after having undergone urogynecological procedures for urinary incontinence, had no problem with their sex life or their relationship with their partner, n = 53; 69.0% and n = 55; 71.4%, respectively. Most of the remaining women reported that incontinence disturbed their sex life somewhat (n = 18; 23.38%), while for the remaining two sets of 3 (3.90%) women each, sex life was disturbed at an average or on a fairly strong level.

In our study, women, after urogynecological procedures for urinary incontinence, indicated discomfort with unpleasant odor at the levels of often, sometimes, and continuously, respectively: n = 24; 31.17%, n = 25; 32.47%, and n = 17; 22.08%. Feelings of shame and embarrassment about bladder dysfunction felt at the levels of sometimes, often, and continuous were reported by n = 30; 38.96%, n = 14; 18.18%, and n = 12; 15.58% of women, respectively. The majority of respondents after urogynecological procedures accepted their condition related to the disease at a good level (n = 43; 55.84%) and 20 (25.97%) women did so at an average (moderate) level. The degree of acceptance of the disease among the surveyed women who underwent urogynecological procedures with urinary incontinence was found to be high.

There was a significant relationship between acceptance of the disease and the quality of life of the surveyed women after urogynecological procedures in the domains studied. Here, the higher the acceptance of the disease, the lower the severity of bladder problems and the higher the quality of life of the subjects. Overall, a strong association between acceptance of the disease and the quality of life for women was obtained for the domain involving emotions. More research, however, is needed to diagnose the problems of women as well as men with incontinence so that preventive strategies can be introduced at the early stages of the disease. Moreover, measures to speed up diagnosis and eliminate factors that affect the quality of life, including mental health, of people with incontinence need to be put into place. Since incontinence is a chronic condition, the prevalence of which is likely to increase as the elderly population grows, it is important to consider not only the economic and social burdens, but also the emotional burden of incontinent patients in care planning.

### Limitations of the Study

Several limitations of this study should be noted. The study did not include a control group of women who did not undergo urogynecological procedures. The women were not asked about the quality of life and acceptance of the illness before the operation, but only after the operation. Moreover, a relatively small group of women was studied, so further research is needed in this area. In addition, it is worth noting that urinary incontinence is an embarrassing topic for women, which may affect their responses and reluctance to participate in such studies.

## 5. Conclusions

Urogynecological procedures in women with urinary incontinence are not always successful and thus do not always improve their quality of life. This applies to domains such as the impact of bladder problems on life and the severity of discomfort. Age and time since urogynecological surgery in women due to urinary incontinence are important in assessing the quality of life. Urogynecological procedures performed in elderly women more often do not bring positive results. There is an intensification of problems related to bladder dysfunction and a lower quality of life.

The longer the time since the procedure, the lower the intensity of negative emotions such as despondency, anxiety, nervousness and negative self-perception and at the same time, the higher the reported quality of life. It is important that every woman with urinary incontinence who is scheduled for surgery, in addition to being presented with alternative surgical options, has the opportunity to receive psychological help after the surgery. Our study showed that the higher the acceptance of the disease, the higher the assessment of the quality of life by women.

## Figures and Tables

**Table 1:** Characteristics of the respondents.

Variables		M	SD
Age		54.46	12.20
Time since surgery		4.35	4.82
Duration of illness		5.04	3.32
		**N**	**%**
Marital status	married	59	76.62%
	widowed	12	15.58%
	divorced	4	5.19%
	single	1	1.30%
	casual relationship	1	1.30%
Type of delivery	natural childbirth	62	80.52%
	cesarean section	11	14.29%
	both natural childbirth and cesarean section	4	5.19%
Type of urinary incontinence	stress urinary incontinence	43	55.84%
	mixed incontinence	20	25.97%
	overactive bladder (urge incontinence)	4	5.19%
Urogynecological procedures	TVT sling	25	32.47%
	TOT sling	23	29.87%
	vaginoplasty	16	20.78%
	colposuspension	6	7.79%
	no specific answer	7	9.09%

**Table 2:** Assessment of the impact of bladder dysfunction on daily life according to the KHQ.

Impact of Bladder Dysfunction on Daily Life as Assessed by Women	N	%
Not at all	10	12.99
Somewhat	22	28.57
Moderate	19	24.68
Very	26	33.77

**Table 3:** Severity of symptoms related to bladder dysfunction according to KHQ.

Exacerbation of Symptoms Related to Bladder Function	None	Somewhat	Moderate	Rather Severe
Frequent use of toilet	18 (23.38%)	13 (16.88%)	38 (49.35%)	8 (10.39%)
Nocturia: getting up during the night to micturate	19 (24.68%)	23 (29.87%)	28 (36.36%)	7 (9.09%)
Sudden urge: a strong and difficult to control urge to urinate	30 (38.96%)	20 (25.97%)	19 (24.68%)	8 (10.39%)
Urge incontinence: urinary leakage associated with a strong need to urinate	25 (32.47%)	16 (20.78%)	23 (29.87%)	13 (16.88%)
Stress urinary incontinence: release of urine with physical activity, including coughing, sneezing, running	21 (27.27%)	23 (29.87%)	12 (15.58%)	21 (27.27%)
Bedwetting at night	49 (63.64%)	16 (20.78%)	9 (11.69%)	3 (3.90%)
Urinary incontinence during intercourse: urinary leakage	55 (71.43%)	13 (16.88%)	8 (1.30%)	1 (1.30%)
Frequent bladder infections	33 (42.86%)	21 (27.27%)	18 (23.38%)	5 (6.49%)
Bladder pain	40 (51.95%)	22 (28.57%)	13 (16.88%)	2 (2.60%)
Urinating difficulty	45 (58.44%)	17 (22.08%)	13 (16.88%)	2 (2.60%)

**Table 4:** The impact of bladder dysfunction on the performance of daily activities, physical and social limitations, personal life, emotions, rest and well-being, and the occurrence of incontinence discomfort according to the KHQ.

The Level and Extent of Limitations in the Performance of Daily Activities in the Subjective Assessment of Respondents	None	Somewhat	Moderate	Rather Severe	
Performing daily household chores such as cleaning, cooking, shopping	33 (42.86%)	27 (35.06%)	12 (15.58%)	5 (6.49%)	
Performing professional duties (work) and other activities outside the home	23 (29.87%)	33 (42.86%)	16 (20.78%)	5 (6.49%)	
**Type and degree of physical and social limitation**	**None**	**Somewhat**	**Moderate**	**Rather severe**	
Impact on physical activity, e.g., walking, running, playing sports, going to the gym, etc.	25 (32.47%)	32 (41.56%)	12 (15.58%)	8 (10.39%)	
Traveling	23 (29.87%)	30 (38.96%)	19 (24.68%)	5 (6.49%)	
Limiting social contacts	45 (58.44%)	15 (19.48%)	12 (15.58%)	5 (6.49%)	
Social gatherings, e.g., friends, acquaintances	48 (62.34%)	18 (23.38%)	8 (10.39%)	3 (3.90%)	
**Personal life**	**Not applicable**	**None**	**Somewhat**	**Moderate**	**Rather severe**
Impact on relationship with partner	26 (33.77%)	29 (37.66%)	16 (20.78%)	4 (5.19%)	2 (2.60%)
Impact on sex life	30 (38.96%)	23 (29.87%)	18 (23.38%)	3 (3.90%)	3 (3.90%)
Impact on family life	17 (22.08%)	38 (49.35%)	15 (19.48%)	5 (6.49%)	2 (2.60%)
**Type and intensity of emotions**	**None**	**Somewhat**	**Moderate**	**Rather severe**	
Sense of despondency	30 (38.96%)	36 (46.75%)	6 (7.79%)	5 (6.49%)	
Sense of uncertainty	26 (33.77%)	37 (48.05%)	6 (7.79%)	8 (10.39%)	
Lowered self-esteem	47 (61.04%)	18 (23.38%)	8 (10.39%)	4 (5.19%)	
**Level of rest and well-being as subjectively assessed by respondents**	**Never**	**Sometimes**	**Often**	**Continuous**	
Sleep disorders	17 (22.08%)	49 (63.64%)	8 (10.39%)	3 (3.90%)	
Feelings of fatigue or exhaustion	28 (36.36%)	38 (49.35%)	10 (12.99%)	1 (1.30%)	
** Type and severity of discomfort as subjectively assessed by subjects **	** Never **	** Sometimes **	** Often **	** Continuous **	
Wearing sanitary pads to keep underwear dry	13 (16.88%)	24 (31.17%)	17 (22.08%)	23 (29.87%)	
Paying special attention to the amount of fluid intake	18 (23.38%)	20 (25.97%)	29 (37.66%)	10 (12.99%)	
The need to change underwear as a result of urine wetting	14 (18.18%)	20 (25.97%)	20 (25.97%)	23 (29.87%)	
Emitting an unpleasant odor	11 (14.29%)	25 (32.47%)	24 (31.17%)	17 (22.08%)	
Feelings of shame and embarrassment due to bladder dysfunction	21 (27.27%)	30 (38.96%)	14 (18.18%)	12 (15.58%)	

**Table 5:** Quality of life assessment according to the KHQ as per the domains studied.

Quality of Life Measured by the KHQ	N	M	SD	Min	Max	Me
General health	77	28.90	19.89	0.0	75.0	25.0
Impact of bladder problems on life	77	59.74	35.18	0.0	100.0	66.67
Limitations in performing daily activities	77	31.60	28.17	0.0	100.0	33.33
Physical limitations	77	35.28	28.09	0.0	100.0	33.33
Social limitations	77	19.48	26.42	0.0	100.0	0.0
Personal life	52	19.23	26.07	0.0	100.0	0.0
Emotions	77	26.26	26.30	0.0	100.0	22.22
Sleep/effort	77	29.22	21.32	0.0	100.0	33.33
Measures of discomfort severity	77	52.81	26.30	0.0	100.0	58.33
Symptom severity scale	77	9.73	5.87	0.0	23.0	10.0

**Table 6:** Relationship between time since surgery and age and the quality of life of respondents as per the domains studied.

Quality of Life Measured by the KHQ	Correlation with Time Since Surgery			Correlation with Age		
	N	R	*p*	N	R	*p*
General health	77	−0.007697	0.947029	77	0.242346	0.033707
Impact of bladder problems on life	77	−0.068215	0.555539	77	−0.213992	0.061655
Limitations in performing daily activities	77	−0.047073	0.684348	77	0.251398	0.027421
Physical limitations	77	−0.051267	0.657909	77	0.141897	0.218316
Social limitations	77	0.029256	0.800599	77	0.179496	0.118282
Personal life	52	0.134097	0.343248	52	0.214453	0.126843
Emotions	77	−0.275918	0.015144	77	0.095812	0.407157
Sleep/effort	77	0.085877	0.457713	77	0.177132	0.123289
Measures of discomfort severity	77	−0.039211	0.734929	77	0.101124	0.381522
Symptom severity scale	77	0.017807	0.877838	77	0.090110	0.435766

**Table 7:** Degree of acceptance of the disease, as measured by the AIS scale, by women who underwent urogynecological procedures for urinary incontinence.

AIS Scale	N	M	SD	Min	Max	Me
Overall score	77	29.06	10.23	8.0	40.0	32.0

**Table 8:** Relationship between acceptance of the disease and the quality of life.

Quality of Life Measured by the KHQ	Correlation with Acceptance Level of the Disease as Measured by the AIS Scale		
	N	R	*p*
General health	77	−0.236060	0.038748
Impact of bladder problems on life	77	−0.153440	0.182759
Limitations in performing daily activities	77	−0.526714	0.000001
Physical limitations	77	−0.554072	<0.00001
Social limitations	77	−0.474735	0.000013
Personal life	52	−0.318305	0.021465
Emotions	77	−0.602849	<0.00001
Sleep/effort	77	−0.496894	0.000004
Measures of discomfort severity	77	−0.563252	0.000000
Symptom severity scale	77	−0.398549	0.000331

## Data Availability

The data presented in this study are available on request from the corresponding author.

## References

[1] Åström Y., Asklund I., Lindam A., Sjöström M. (2021). Quality of life in women with urinary incontinence seeking care using e-health. BMC Womens Health.

[2] Elenskaia K., Haidvogel K., Heidinger C., Doerfler D., Umek W., Hanzal E. (2011). The greatest taboo: Urinary incontinence as a source of shame and embarrassment. Wien Klin Wochenschr..

[3] Bardsley A. (2016). An overview of urinary incontinence. Br. J. Nurs..

[4] Schreiber Pedersen L., Lose G., Høybye M.T., Jürgensen M., Waldmann A., Rudnicki M. (2017). Predictors and reasons for help-seeking behavior among women with urinary incontinence. Int. Urogynecol. J..

[5] Gümüşsoy S., Kavlak O., Donmez S. (2019). Investigation of body image, self-esteem, and quality of life in women with urinary incontinence. Int. J. Nurs. Pr..

[6] Corrado B., Giardulli B., Polito F., Aprea S., Lanzano M., Dodaro C. (2020). The Impact of Urinary Incontinence on Quality of Life: A Cross-Sectional Study in the Metropolitan City of Naples. Geriatrics.

[7] Steibliene V., Aniuliene R., Aniulis P., Raskauskiene N., Adomaitiene V. (2020). Affective Symptoms and Health-Related Quality of Life Among Women with Stress Urinary Incontinence: Cross-Sectional Study. Neuropsychiatr. Dis. Treat..

[8] Moran P.A., Dwyer P.L., Ziccone S.P. (1999). Urinary leakage during coitus in women. J. Obstet. Gynaecol..

[9] Coyne K.S., Sexton C.C., Irwin D.E., Kopp Z.S., Kelleher C.J., Milsom I. (2008). The impact of overactive bladder, incontinence and other lower urinary tract symptoms on quality of life, work productivity, sexuality and emotional well-being in men and women: Results from the EPIC study. BJU Int..

[10] Özdemir K., Şahin S., Özerdoğan N., Ünsal A. (2018). Evaluation of urinary incontinence and quality of life in married women aged between 20 and 49 years (Sakarya, Turkey). Turk. J. Med. Sci..

[11] Kwon B.E., Kim G.Y., Son Y.J., Roh Y.S., You M.A. (2010). Quality of life of women with urinary incontinence: A systematic literature review. Int. Neurourol. J..

[12] Pizzol D., Demurtas J., Celotto S., Maggi S., Smith L., Angiolelli G., Trott M., Yang L., Veronese N. (2021). Urinary incontinence and quality of life: A systematic review and meta-analysis. Aging Clin. Exp. Res..

[13] Hashim H., Blanker M.H., Drake M.J., Djurhuus J.C., Meijlink J., Morris V., Petros P., Wen J.G., Wein A. (2019). International Continence Society (ICS) report on the terminology for nocturia and nocturnal lower urinary tract function. Neurourol. Urodyn..

[14] Lukacz E.S., Santiago-Lastra Y., Albo M.E., Brubaker L. (2017). Urinary Incontinence in Women: A Review. JAMA.

[15] Martínez Agulló E., Ruíz Cerdá J.L., Gómez Pérez L., Rebollo P., Pérez M., Chaves J. (2010). Impact of urinary incontinence and over-active bladder syndrome on health-related quality of life of working middle-aged patients and institutionalized elderly patients. Actas Urol. Esp.

[16] Post M.W. (2014). Definitions of quality of life: What has happened and how to move on. Top Spinal Cord Inj. Rehabil..

[17] Senra C., Pereira M.G. (2015). Quality of life in women with urinary incontinence. Rev. Assoc. Med. Bras. (1992).

[18] Fernandes S., Carvalho Coutinho E., Carvalho Duarte J., Batista Nelas P.A., Correia Balula Chaves C.M., Amaral O. (2015). Quality of life in women with Urinary Incontinence. J. Nurs..

[19] Mikuš M., Kalafatić D., Vrbanić A., Šprem Goldštajn M., Herman M., Živković Njavro M., Živković K., Marić G., Ćorić M. (2022). Efficacy Comparison between Kegel Exercises and Extracorporeal Magnetic Innervation in Treatment of Female Stress Urinary Incontinence: A Randomized Clinical Trial. Medicina (Kaunas).

[20] Frutos-Reoyo E.J., Luque-Linero P., Cantalapiedra-Puentes E., Mendi-Gabarain I., Bermejo-de la Fuente P., Candau-Pérez E.D. (2023). Factores pronósticos del resultado del tratamiento rehabilitador en la incontinencia urinaria femenina. Actas Urológicas Españolas.

[21] Riemsma R., Hagen S., Kirschner-Hermanns R., Norton C., Wijk H., Andersson K.E., Chapple C., Spinks J., Wagg A., Hutt E. (2017). Can incontinence be cured? A systematic review of cure rates. BMC Med..

[22] Kelleher C.J., Cardozo L.D., Khullar V., Salvatore S. (1997). A new questionnaire to assess the quality of life of urinary incontinent women. Br. J. Obstet. Gynaecol.

[23] Felton B.J., Revenson T.A., Hinrichsen G.A. (1984). Stress and coping in the explanation of psychological adjustment among chronically ill adults. Soc. Sci. Med..

[24] Skorupska K., Grzybowska M.E., Kubik-Komar A., Rechberger T., Miotla P. (2021). Identification of the Urogenital Distress Inventory-6 and the Incontinence Impact Questionnaire-7 cutoff scores in urinary incontinent women. Health Qual Life Outcomes.

[25] Malik R.D., Hess D.S., Christie A., Carmel M.E., Zimmern P.E. (2019). Domain Comparison Between 6 Validated Questionnaires Administered to Women With Urinary Incontinence. Urology.

[26] Kieres P., Skorupska K., Mlodawski J., Misiek M., Rokita W., Rechberger T. (2021). Reliability of The King’s Health Questionnaire and the International Consultation on Incontinence Modular Questionnaire (ICIQ-SF) Short Form in assessing urinary incontinence effects in Polish women. Ginekol. Pol..

[27] Wróbel A., Skorupska K., Rechberger E., Woźniak A., Miotla P., Kubik-Komar A., Skorupski P., Rechberger T. (2019). Reliability of the Polish version of the Overactive Bladder Symptom Score (OABSS) questionnaire: Correlation of the OABSS with urodynamic study and the UDI-6 and IIQ-7 questionnaires. Int. Urogynecol. J..

[28] Mikuš M., Ćorić M., Matak L., Škegro B., Vujić G., Banović V. (2020). Validation of the UDI-6 and the ICIQ-UI SF—Croatian version. Int. Urogynecol. J..

[29] Brandt F., Solomayer E.F., Sklavounos P. (2022). Correlation between the Incontinence Severity Index (ISI) and the quality of life dimensions of the King’s Health Questionnaire (KHQ) in German-speaking urinary incontinent women. J. Gynecol. Obstet. Hum Reprod..

[30] Minassian V.A., Devore E., Hagan K., Grodstein F. (2013). Severity of urinary incontinence and effect on quality of life in women by incontinence type. Obstet. Gynecol..

[31] Burzyński B., Kwiatkowska K., Sołtysiak-Gibała Z., Bryniarski P., Przymuszała P., Wlaźlak E., Rzymski P. (2021). Impact of stress urinary incontinence on female sexual activity. Eur. Rev. Med. Pharmacol. Sci..

[32] Nilsson M., Lalos O., Lindkvist H., Lalos A. (2011). How do urinary incontinence and urgency affect women’s sexual life?. Acta Obstet. Et Gynecol. Scand..

